# Fatty acids from dairy and meat and their association with risk of coronary heart disease

**DOI:** 10.1007/s00394-018-1811-1

**Published:** 2018-08-30

**Authors:** Linda E. T. Vissers, Jonna Rijksen, Jolanda M. A. Boer, W. M. Monique Verschuren, Yvonne T. van der Schouw, Ivonne Sluijs

**Affiliations:** 1 Julius Center for Health Sciences and Primary Care, University Medical Center Utrecht, Utrecht University, STRT 6.131, PO Box 85500, 3508 GA Utrecht, The Netherlands; 2 National institute for Public Health and the Environment (RIVM), Bilthoven, The Netherlands

**Keywords:** Fatty acids, Substitution, Replacement, Dairy, Meat, Prospective

## Abstract

**Purpose:**

The relationship of total, saturated, mono-unsaturated and poly-unsaturated fatty acids (SFA, MUFA, PUFA) with coronary heart disease (CHD) is debated. We hypothesized that the association of dairy-derived FA with CHD may be different than the association of meat-derived FA with CHD. We therefore aimed to directly compare association of FA intakes from dairy and meat with risk of CHD using substitution models.

**Methods:**

Baseline (1993–1997) FA intake was measured using a validated food frequency questionnaire among 35,767 participants from the European Prospective Investigation into Cancer and Nutrition-Netherlands cohort (EPIC-NL). Incident CHD events (*n* = 2374) were obtained through linkage with national registries during a mean follow-up of 15 years. Association of FA from dairy substituted with FA from meat with CHD risk was estimated through multivariable Cox regression.

**Results:**

Participants consumed 81.9 (SD 28.7) grams of FA per day, of which 17.9 (SD 5.2) was from dairy and 15.3 (SD 9.5) from meat. Substituting 1 en% of dairy-derived SFA with meat-derived SFA was associated with higher CHD risk (HR 1.06, 95% CI 1.02–1.10), but substituting dairy-derived MUFA or PUFA did not (HR_MUFA_ 1.03, 95% CI 0.97–1.09; HR_PUFA_ 1.17, 95% CI 0.90–1.53).

**Conclusions:**

Our modelling suggests that substituting dairy SFA with meat SFA is associated with a higher risk of CHD, but substituting dairy MUFA or PUFA with meat FA is not. These results need to be replicated in other cohorts with different fat intakes, preferably with larger variation in the intake of MUFA and PUFA from dairy and meat.

**Electronic supplementary material:**

The online version of this article (10.1007/s00394-018-1811-1) contains supplementary material, which is available to authorized users.

## Introduction

Dietary guidelines recommend to keep intake of saturated fatty acids (SFA) below 10% of total energy intake, and to replace SFA by mono-unsaturated fatty acids (MUFA) or poly-unsaturated fatty acids (PUFA) [37]. This recommendation is supported by a large-scale meta-analysis that shows that SFA replacement by *cis*-MUFA or *cis*-PUFA leads to a more favourable lipid profile [18]. However, SFA replacement by MUFA does not clearly reduce CHD risk in observational studies [11, 13, 19]. Observational studies and RCTs suggest that replacement of SFA by PUFA decreases CHD risk [13, 20], although some observational cohort studies did not confirm this [25, 26]. It remains unclear why the substitution of SFA with unsaturated FA does not improve cardiovascular health in all populations.

One hypothesis is that the association of FA with CHD is different, depending on the food source from which it is derived. For instance, dairy intake is associated with CHD risk in a protective or neutral manner [2, 31], whereas meat intake, particularly red or processed meat, relates to a higher risk of CHD [2]. In a Dutch population, SFA from dairy has been related with a lower CHD risk, whereas SFA from meat or fats was not related to CHD [25].

Direct comparison of FA from dairy and meat, in relation to risk of CHD, is needed to gain insight into these differences. This can be achieved by modelling substitution of a specified FA subtype (e.g., SFA) derived from dairy by the same FA subtype derived from meat. When including energy from carbohydrates, protein and all other FA, plus total energy in the regression model, the results from such an analysis can be interpreted as the effect of increasing FA intake from meat at the expense of FA intake from dairy [39].

According to our knowledge, only the multi-ethic study of atherosclerosis (MESA) investigated replacement of dairy-derived SFA with meat-derived SFA, and found that this substitution was related to higher CHD risk [5]. This study was performed in the USA, where consumption patterns of meat and dairy are different from those in Europe [23]. Additionally, replacements of MUFA and PUFA were not addressed in MESA.

Therefore, the aim of this study was to investigate the association of substituting FA from dairy products (SFA, MUFA or PUFA), with FA from meat with CHD risk in a Dutch population that consumes high amounts of dairy and meat products.

## Methods

The European Prospective Investigation into Cancer and Nutrition (EPIC)-NL cohort (*n* = 40,011) consists of the Prospect-EPIC cohort and the Monitoring Project on Risk Factors for Chronic Diseases (MORGEN)-EPIC cohort. These cohorts were set up simultaneously between 1993 and 1997. The Prospect cohort consists of 17,357 women aged 49–70, living in Utrecht or its vicinity, who participated in a Dutch breast cancer screening program. The MORGEN cohort consists of 22,654 men and women, aged 20–59 who were recruited through age stratified random samples of three Dutch towns (Amsterdam, Doetinchem and Maastricht).

All participants provided written informed consent before study inclusion. The study complied with the Declaration of Helsinki and was approved by the medical ethics committee of the Netherlands Organization for Applied Scientific Research (TNO) (MORGEN), and the institutional review board of the University Medical Centre Utrecht (Prospect). Detailed description of the design and rationale of this cohort can be found elsewhere [3]. This manuscript has been written according to the STROBE-nut guideline [16].

We included participants that gave permission for linkage with vital status and disease/medical/mortality registries (*n* = 38,260). Participants with missing data on dietary intake, educational status, BMI, smoking or hypercholesterolemia were excluded (*n* = 516), as were participants with prevalent CVD (*n* = 1,336), participants with potential under- or over-reported energy intake (energy intake/basal metabolic rate in lowest or upper 0.5%) (*n* = 333), and non-consumers of meat or dairy (*n* = 308), leaving a total of 35,767 participants for analysis in this study.

### Assessment of food consumption

The EPIC-NL cohort used a self-administered validated food frequency questionnaire (FFQ) to assess the food consumption (in times per day, per week, per month or per year, or as never) of 79 main food categories during the year before enrolment. The questionnaire also contained colour photographs with portion sizes of 21 foods. The FFQ gives an estimation of the average daily consumption of 178 food items [3, 22]. Nutrient intakes were calculated using the Dutch food composition table of 1996 [34]. Use of dietary supplements was not registered.

We calculated FA intake from dairy, meat and other sources, by multiplying the FA content of whole food items with the daily average consumption of these whole foods. The meat food group included red and processed meat, meat products and poultry. The dairy food group included cheese, milk, yoghurt, coffee creamers, curd, pudding, porridge, custard, and whipping cream (Supplemental Table 1).

Validity of the FFQ was assessed by comparison with 12 24 h recalls among 121 men and women. In men, Spearman’s rank correlation coefficients showed good validity for milk and milk products (0.69), moderate validity for cheese (0.56), fair validity for meat (0.39) [22] and good to moderate validity for the intake of FA (SFA 0.55, MUFA 0.66, PUFA 0.52) [24]. In women, the validity was good for milk and milk products (0.77), fair for cheese (0.32), moderate for meat (0.59) [22] and moderate to fair for the intake of FA (SFA 0.50, MUFA 0.58, PUFA 0.22) [24].

### Assessment of covariates

A general questionnaire was administered at baseline, which included questions on demographic characteristics and cardiovascular risk factors. Educational level was categorized into three groups: low (primary till intermediate vocational education), moderate (higher general secondary education completed or till 3rd year with success), and high (higher vocational education and university). Smoking status was categorized as never, former, or current smoker. Physical activity was assessed using a validated questionnaire [12]. The Cambridge Physical Activity Score [36] was then calculated and used to categorize physical activity into inactive, moderately inactive, moderately active and active. Participants in the latter two categories were considered to be physically active. Because we could not calculate the Cambridge physical activity score for 14% of all participants, we imputed missing scores by means of single linear regression modelling (SPSS MVA procedure). Presence of hypercholesterolemia was self-reported. Daily alcohol consumption (g/day) was categorized into four groups: light drinkers (0–4.9 g/day), moderate drinkers (5–14.9 g/days), heavy drinkers (15–29.9 g/day), and excessive drinkers (> 30 g/day).

Furthermore, a physical examination was performed. Body weight (kg) and height (cm) were measured and BMI was calculated by dividing weight (kg) by height (m) squared (kg/m^2^). Waist and hip circumference (cm) were measured twice, and waist-to-hip ratio (WHR) was calculated by dividing the mean waist by mean hip circumference. Systolic- and diastolic blood pressure (mmHg) were measured twice on participants’ left arm, while in the supine position, with a Boso Oscillomat (Prospect) or with a random-zero sphygmomanometer (MORGEN). The mean of these two measurements was used. Hypertension was defined when one of the following criteria was met: systolic blood pressure > 140 mmHg; diastolic blood pressure > 90 mmHg; self-reported use of antihypertensive medication, or self-reported physician-diagnosed hypertension.

### Assessment of coronary heart disease

To obtain cases of incident CHD (fatal and non-fatal CHD combined), participants were followed over time by linkage to mortality and hospital discharge registers. Data on hospital discharge diagnoses were provided by the National Medical Registry, using the Dutch Hospital Discharge Diagnosis Database. Vital status was obtained through linkage with the municipal population registries. Primary and secondary causes of death were obtained from Statistics Netherlands.

In the Dutch Hospital Discharge Diagnosis Database, CHD was defined through International Classification of Diseases (ICD)-9 codes: 410-414, 427.5, 798.1, 798.2, and 798.9. Causes of death were coded according to ICD-10: I-20-I25, I46, and R96.

### Data analysis

Participants’ baseline characteristics were explored by tertiles of FA intake from dairy and meat in percentage of total energy intake (en%). Results are presented as percentage for categorical variables, mean ± standard deviation (SD) for continuous variables and median (interquartile range) for continuous variables that are not normally distributed. We examined intake of total fat, SFA, MUFA, and PUFA from dairy sources, and from meat sources separately. We examined the relative contributions of individual SFA to the total SFA intake from dairy sources and meat sources as well. Pearson correlations between intake of total fat, SFA, MUFA and PUFA (in en%) from dairy sources, and from meat sources were calculated. The percentage of SFA, MUFA and PUFA intake from various sources is calculated as well.

Person-years of follow-up were calculated from date of study inclusion to date of first CHD event, death, loss to follow-up, or end of follow-up (December 31th 2010), whichever came first. We used Cox proportional regression analysis to estimate Hazard Ratios (HRs) and 95% confidence intervals (CIs) for the association between substituting FA (total, SFA, MUFA, PUFA) from dairy by FA from meat, and risk of CHD.

We created a crude substitution model in which 1% of energy intake (1 en%) from meat FA was replaced by 1en% from dairy FA, by including total energy intake (excluding energy from alcohol), and energy from macronutrients (protein, carbohydrates, FA including trans fats). The only macronutrient not included in the model was the dairy FA that was to be replaced by the corresponding meat FA. For example, this means that a substitution model of dairy SFA with meat SFA includes protein, carbohydrates, all MUFAs, all PUFAs and SFA from meat and from other sources than meat or dairy. The estimate from this model can be interpreted as the association of consuming 1en% SFA from meat instead of 1en% SFA from dairy with risk of CHD.

We applied three models of adjustment for potential confounding. We considered known risk factors for CHD and covariates that were associated with FA intake and CHD risk in our population. Model 1 was adjusted for sex and age. Model 2 was additionally adjusted for smoking, physical activity, educational level, alcohol intake (in categories) and energy-adjusted intake of cholesterol and fibre, calculated using the residual method [38]. Our final model, model 3, was adjusted for cardiovascular risk factors that might also be an intermediate such as BMI, hypercholesterolemia, type 2 diabetes and hypertension. These can be intermediates for the substitution of FA from dairy with FA from meat, because a high intake of dairy has been associated with a lower BMI [15] and a lower risk of diabetes [10] and hypertension [30, 32]. In contrast, a high intake of meat has been associated with higher BMI [29, 35] and a higher risk of hypertension [30] and diabetes [1].

We performed aforementioned analyses four times; for the substitution of total fat, SFA, MUFA, and PUFA.

A previous study has suggested possible effect modification of the relation between dietary fat and CHD by sex and age [14], which we have examined by including an interaction term between the determinant and sex or age in the final substitution model. Non-linearity was examined by including quadratic terms of the FA of interest into the final adjusted model. Whether hazards are constant over time was examined by adding an interaction term between determinant and time.

For comparison, we also performed Cox regression models for the increase of FA from dairy or meat, without specifying a substituting macronutrient. We created two basic models, one adjusting for energy and protein intake, and the other adjusting for energy and carbohydrate intake. We show results for models that have been adjusted for covariates from models 2 and 3. Estimates from these models can be interpreted as the combined effect of increasing energy from dairy or meat FA, and lowering intake of another (unspecified) macronutrient.

We repeated the analyses by adjusting for WHR, instead of BMI. Possible reverse causation was examined by repeating analyses after excluding the first two years of follow-up. All analyses were performed using Statistical Package for the Social Sciences (SPSS) version 23.

## Results

At baseline, the average fat intake in the EPIC-NL cohort was 81.9 ± 28.7 g/day, which is 35.6 ± 5.2 en%/day. Of this total fat intake, 17.9 ± 9.5 g/day (7.7 ± 3.5 en%/day) was derived from dairy, and 15.3 ± 9.5 g/day (6.5 ± 3.5 en%/day) from meat. Average SFA intake was 34.4 ± 12.4 g/day (14.6 ± 2.5 en%/day) in total, 11.5 ± 6.0 g/day (4.9 ± 2.2 en%/day) from dairy, and 6.0 ± 3.7 g/day (2.6 ± 1.4 en%/day) from meat. The main sources of SFA and MUFA were meat (21% and 29%), cheese (19% and 11%), and milk products (19% and 9%). PUFA was mainly derived from bread/cereals (26%), meat (14%), and savoury sauces (9%). Approximately 6% of PUFA was derived from cheese and milk products combined (Supplementary Fig. 1).

Participants with a high intake of dairy-derived total fat were more often women at an older age, highly educated, physically active, light drinkers and non-smokers when compared to participants with a low intake. They were more likely to have hypertension or type 2 diabetes, but less likely to smoke or have hypercholesterolemia. Participants with a high dairy-derived fat intake also reported lower intake of meat and soft drinks, and a higher intake of fruit and vegetables (Table 1). Participants with a high intake of meat-derived fat were generally less healthy (Table 2). Baseline characteristics by tertiles of SFA, MUFA and PUFA from dairy and meat are similar to characteristics by tertiles of total fat (Supplemental Tables 2–7).

**Table 1:** Baseline characteristics of 35,767 EPIC-NL cohort participants per tertile of dairy-derived total fat intake (in % of total energy intake minus energy from alcohol)

	En% of dairy-derived total fat		
	*T*_1_ (< 6.0)	*T*_2_ (6.1–8.9)	*T*_3_ (8.9–37.1)
Participants (*n*)	11,922	11,923	11,923
Women (%)	64.2	76.7	83.6
Age (years)	45.8 ± 12.4	49.8 ± 11.6	51.9 ± 10.7
BMI (kg/m^2^)	25.6 ± 4.0	25.7 ± 3.9	25.6 ± 4.0
WHR	0.83 ± 0.09	0.82 ± 0.09	0.81 ± 0.08
High education level^a^ (%)	18.0	20.1	22.9
Light alcohol consumption^b^ (%)	49.1	49.9	50.4
Physically active (%)	40.9	41.9	43.1
Smoking			
Former smoker (%)	28.9	32.2	35.6
Current smoker (%)	34.8	28.3	28.1
Hypertension (%)	34.5	38.0	37.9
Hypercholesterolemia (%)	9.6	8.0	5.9
Type 2 diabetes (%)	1.1	1.2	1.7
Energy intake (kcal/day)	2118 ± 655	2056 ± 584	1988 ± 568
Dairy products (g/day)	286 ± 211	452 ± 245	566 ± 310
Dairy fat (en%/day)			
Total fat	4.2 ± 1.4	7.4 ± 0.8	11.6 ± 2.5
SFA	2.7 ± 0.9	4.8 ± 0.5	7.4 ± 1.6
MUFA	1.2 ± 0.4	2.2 ± 0.3	3.5 ± 0.8
PUFA	0.2 ± 0.1	0.3 ± 0.1	0.5 ± 0.2
Meat products (g/day)	127 ± 61	107 ± 51	90 ± 50
Meat fat (en%/day)			
Total fat	7.5 ± 3.8	6.5 ± 3.3	5.5 ± 3.2
SFA	2.9 ± 1.5	2.6 ± 1.3	2.2 ± 1.3
MUFA	3.5 ± 1.8	3.0 ± 1.5	2.6 ± 1.5
PUFA	0.9 ± 0.5	0.7 ± 0.4	0.6 ± 0.4
Protein (en%/day)	15.0 ± 2.4	16.1 ± 2.2	17.1 ± 2.2
Carbohydrates (en%/day)	48.4 ± 5.9	47.1 ± 5.3	45.0 ± 5.4
Fat (en%/day)	36.6 ± 5.6	36.8 ± 5.1	37.8 ± 5.1
Trans fat (en%/day)	1.5 ± 0.6	1.4 ± 0.5	1.4 ± 0.4
Soft drinks (ml/day)	131 ± 164	95 ± 125	75 ± 107
Fruit and vegetables (g/day)	382 ± 194	409 ± 182	404 ± 175
Fish (g/day)	10 ± 11	10 ± 11	10 ± 10
Nuts and seeds (g/day)	8 ± 13	7 ± 11	6 ± 9
Coffee and tea (ml/day)	809 ± 380	858 ± 335	884 ± 348
Cholesterol^c^ (mg/day)	208 ± 61	216 ± 56	230 ± 56
Fibre^c^ (g/day)	23 ± 5	24 ± 5	23 ± 5

Data is presented as mean ± SD or as percentage

*WHR* waist-to-hip ratio, *En%* percentage of total energy intake, *MUFA* mono-unsaturated fatty acid, *PUFA* poly-unsaturated fatty acid, *SFA* saturated fatty acid

^a^Higher vocational education and university

^b^< 4.9 g of alcohol per day

^c^Energy adjusted

**Table 2:** Baseline characteristics of 35,767 EPIC-NL cohort participants per tertile of meat-derived total fat intake (in % of total energy intake minus energy from alcohol)

	En% of meat-derived total fat		
	*T*_1_ (< 4.7)	*T*_2_ (4.7–7.6)	*T*_3_ (7.6–51.2)
Participants (*n*)	11,922	11,923	11,922
Women (%)	82.0	73.1	69.4
Age (years)	50 ± 12	49 ± 12	49 ± 11
BMI (kg/m2)	24.8 ± 3.7	25.7 ± 3.9	26.5 ± 4.1
WHR	0.80 ± 0.08	0.82 ± 0.09	0.84 ± 0.09
High education level^a^ (%)	27.3	19.9	13.8
Light alcohol consumption ^b^ (%)	54.6	48.7	46.0
Physically active (%)	43.1	42.9	40.0
Smoking			
Former smoker (%)	31.9	31.2	30.6
Current smoker (%)	21.6	29.5	35.5
Hypertension (%)	34.4	36.5	39.4
Hypercholesterolemia (%)	7.2	7.5	8.8
Type 2 diabetes (%)	1.1	1.2	1.7
Energy intake (kcal/day)	1991 ± 601	2111 ± 611	2059 ± 600
Dairy products (g/day)	496 ± 309	447 ± 273	361 ± 246
Dairy fat (en%/day)			
Total fat	8.8 ± 3.8	7.6 ± 3.2	6.8 ± 3.2
SFA	5.7 ± 2.4	4.9 ± 2.0	4.4 ± 2.0
MUFA	2.7 ± 1.2	2.3 ± 1.0	2.0 ± 1.0
PUFA	0.4 ± 0.2	0.3 ± 0.2	0.3 ± 0.2
Meat products (g/day)	58 ± 33	112 ± 36	153 ± 50
Meat fat (en%/day)			
Total fat	2.9 ± 1.2	6.1 ± 0.8	10.4 ± 2.6
SFA	1.2 ± 0.5	2.4 ± 0.3	4.1 ± 1.0
MUFA	1.4 ± 0.6	2.9 ± 0.4	4.9 ± 1.2
PUFA	0.3 ± 0.1	0.7 ± 0.2	1.2 ± 0.4
Protein (en%/day)	15.4 ± 2.4	16.1 ± 2.3	16.8 ± 2.4
Carbohydrates (en%/day)	49.8 ± 5.4	47.1 ± 4.8	43.7 ± 5.1
Fat (en%/day)	34.8 ± 5.3	36.8 ± 4.7	39.5 ± 4.8
Trans fat (en%/day)	1.4 ± 0.5	1.5 ± 0.5	1.5 ± 0.5
Soft drinks (ml/day)	88 ± 128	105 ± 135	108 ± 145
Fruit and vegetables (g/day)	440 ± 198	398 ± 176	356 ± 168
Fish (g/day)	11 ± 13	10 ± 10	9 ± 9
Nuts and seeds (g/day)	8 ± 13	7 ± 11	5 ± 9
Coffee and tea (ml/day)	848 ± 362	848 ± 345	855 ± 361
Cholesterol^c^ (mg/day)	195 ± 57	218 ± 53	241 ± 56
Fibre^c^ (g/day)	24 ± 5	23 ± 5	22 ± 5

Data is presented as mean ± SD or as percentage

*WHR* waist-to-hip ratio, *En%* percentage of total energy intake, *MUFA* mono-unsaturated fatty acid, *PUFA* poly-unsaturated fatty acid, *SFA* saturated fatty acid

^a^Higher vocational education and university

^b^< 4.9 g of alcohol per day

^c^Energy adjusted

In EPIC-NL, most fat derived from dairy was SFA (65.2%), followed by MUFA (30.6%) and PUFA (4.2%). Most fat derived from meat was MUFA (47.9%), followed by SFA (40.3%) and PUFA (11.8%). Stearic acid (C18:0) and palmitic acid (C16:0) were the most common individual SFAs consumed and consumption of other individual SFAs from dairy and meat was low (< 2.5%) (Table 3). Intakes of total fat, SFA, MUFA and PUFA from meat sources were highly correlated (Pearson’s rho > 0.80). The same was found for fats from dairy sources (Table 4).

**Table 3:** Contribution of individual fatty acids to total saturated fatty acid intake from dairy and meat among 35,767 EPIC-NL participants

Saturated fat type	Dairy	Meat
C14:0 myristic acid	2.3%	< 0.5%
C15:0 pentadecyclic acid	1.1%	< 0.5%
C16:0 palmitic acid	5.7%	5.4%
C17:0 margaric acid	1.1%	0.6%
C18:0 stearic acid	89.7%	91.6%
C20:0 arachidic acid	< 0.5%	2.1%
C22:0 behenic acid	–	< 0.5%
C24:0 lignoceric acid	< 0.5%	–

Data are expressed as percentage of total SFA intake from food source

**Table 4:** Correlation between fatty acids within meat products and between fatty acids within dairy products in 35,767 participants from the EPIC-NL cohort, adjusted for sex and age at recruitment

Dairy	Total fat	SFA	MUFA	PUFA
Total fat	1			
SFA	1.00	1		
MUFA	0.99	0.98	1	
PUFA	0.83	0.79	0.85	1

Meat	Total fat	SFA	MUFA	PUFA
Total fat	1			
SFA	1.00	1		
MUFA	1.00	1.00	1	
PUFA	0.96	0.94	0.95	1

Data are Pearson’s rho correlation coefficients (*Ῥ*) for correlation of FA expressed as % of total energy intake within whole food, adjusted for sex and age at recruitment. All *P* values are < 0.001

*EPIC-NL* European Prospective Investigation into Cancer and Nutrition-Netherlands, *SFA* saturated fatty acids, *MUFA* mono-unsaturated fatty acids, *PUFA* poly-unsaturated fatty acids

A total of 2458 participants developed CHD among the 35,767 participants during a median follow-up time of 15 years. Substituting 1en% of total FA and SFA from dairy with 1en% corresponding FA from meat was associated with a higher risk of CHD in model 2 (corrected for demographic and cardiovascular risk factors), but substitution of MUFA or PUFA was not (total fat 3%, 95% CI 2 to 5%; SFA 8%, 95% CI 4 to 13%; MUFA 4%, 95% CI − 1 to 10%, PUFA 20%, 95%CI − 7 to 57%). After further adjustment for cardiovascular risk factors that are possible intermediates (BMI, hypertension, type 2 diabetes, hypercholesterolemia), results were attenuated (total fat 2%, 95% CI 0–4% ; SFA 6%, 95% CI 2–10%; MUFA 3%, 95% CI − 7 to 9%, PUFA 17%, 95%CI − 10 to 53%), but remained significant for the substitution of SFA (Table 5).

**Table 5:** Hazard of CHD when substituting dairy-derived FA intake by meat-derived FA intake at baseline among 35,767 EPIC-NL participants

	Substitution model 1	Substitution model 2	Substitution model 3
Total fat	1.04 (1.03–1.06)	1.03 (1.02–1.05)	1.02 (1.00–1.04)
SFA	1.14 (1.09–1.18)	1.08 (1.04–1.13)	1.06 (1.02–1.10)
MUFA	1.07 (1.02–1.14)	1.04 (0.99–1.10)	1.03 (0.97–1.09)
PUFA	1.10 (0.86–1.40)	1.20 (0.93–1.57)	1.17 (0.90–1.53)

The substitution model was corrected for total caloric intake except from alcohol, intake of protein, carbohydrates and all FA, except for dairy-derived FA from the substitution of interest (SFA, MUFA or PUFA). Substitution model 1 was adjusted for sex and age. Model 2 was additionally adjusted for smoking, physical activity, education, alcohol intake (categorical) and energy-adjusted intake of cholesterol and fibre. Model 3 was additionally adjusted for BMI, hypertension, type 2 diabetes and hypercholesterolemia

After excluding the first 2 years of follow-up, results did not change materially. Adjusting for WHR instead of BMI did not change results. We did not find evidence for a non-linear association between any FA substitution and risk of CHD, nor did we find evidence for an interaction with sex or age. Hazards were constant over time.

In the protein or carbohydrates intake adjusted models where we did not specify a substituting macronutrient (Table 6), FA from meat were consistently associated with a higher CHD risk in the final adjusted model, whereas FA from dairy were not. 

**Table 6:** Hazard of CHD per 1en% increase of meat or dairy FA, without specifying the replacing macronutrient among 35,767 EPIC-NL participants

	Protein model^a^ 2	Protein model 3	Carbohydrates model 2	Carbohydrates model 3
Meat				
Total fat	1.02 (1.01–1.04)	1.02 (1.01–1.03)	1.03 (1.01–1.04)	1.02 (1.01–1.03)
SFA	1.06 (1.03–1.10)	1.04 (1.01–1.08)	1.07 (1.04–1.11)	1.05 (1.01–1.08)
MUFA	1.05 (1.03–1.08)	1.04 (1.01–1.06)	1.06 (1.03–1.09)	1.04 (1.01–1.07)
PUFA	1.17 (1.07–1.28)	1.11 (1.01–1.21)	1.18 (1.07–1.30)	1.11 (1.01–1.23)
Dairy				
Total fat	0.98 (0.97–1.00)	0.99 (0.98–1.00)	0.99 (0.98–1.00)	0.99 (0.98–1.00)
SFA	0.97 (0.95–0.99)	0.98 (0.96–1.00)	0.98 (0.96–1.00)	0.99 (0.97–1.01)
MUFA	0.94 (0.91–0.99)	0.97 (0.93–1.01)	0.96 (0.92–1.00)	0.97 (0.93–1.01)
PUFA	0.85 (0.66–1.08)	0.91 (0.71–1.16)	0.92 (0.72–1.16)	0.92 (0.72–1.16)

Model 2 was adjusted for sex, age, smoking, physical activity, education, alcohol intake (categorical), energy-adjusted intake of cholesterol and fibre, intake of trans fats (en%) and total energy intake (excluding energy from alcohol). Model 3 was additionally adjusted for BMI, hypertension, type 2 diabetes and hypercholesterolemia

^a^For protein models, intake of protein (en%) was added to model 2 and 3, whereas carbohydrates models included intake of carbohydrates (en%)

## Discussion

In this prospective cohort study among 35,767 men and women, we observed that substituting 1en% SFA from dairy with SFA from meat was associated with a 6% higher risk of coronary heart disease. Although effect estimates suggest a similar association for MUFA and PUFA, the association for substitution of MUFA or PUFA from dairy with the corresponding FA from meat was not statistically significant. In the models where substitution was not specified, consuming more FA from meat was associated with a higher risk of CHD, whereas a higher FA intake from dairy was generally not associated with incident CHD risk.

We modelled the substitution of SFA from dairy with SFA from meat per 1 en%. For women, based on the recommended energy intake of 2000 kcal/day, this would require substituting 222 g/day of semi-skimmed milk with 147 g/day of prepared lean beef [34]. Results from this study cannot be interpreted on the level of individual whole foods, because they are based on the SFA contribution from a group of dairy and meat products. However, the dietary replacement in terms of whole foods does exemplify that implementing the substitution of 1en% SFA from dairy with SFA from meat would require a substantial change in dietary habits.

The effect direction of our results is in line with findings from previous studies. Substituting 2 en% of dairy-derived SFA with 2 en% of meat-derived SFA has been associated with a 25% higher risk of CHD in the MESA study [5]. Consistently, substituting 5 en% of dairy FA with non-dairy animal FA has been associated with a 6% higher risk of CHD in three cohorts of US adults [4]. Substituting MUFA or PUFA from dairy with MUFA or PUFA from meat has not been investigated to date.

Results from the models where substitution was not specified suggest that MUFA and PUFA from meat increases CHD risk, whereas MUFA and PUFA from dairy does not. These differences seem substantial, as the confidence intervals for the association of MUFA intake and CHD risk from meat versus dairy do not overlap. However, our substitution analysis suggests that replacing MUFA from dairy with MUFA from meat is not related to CHD risk. Modelling substitution in populations with a larger spread in overlapping MUFA and PUFA intake from meat and dairy is necessary to exclude the possibility of insufficient power of the substitution analysis as a cause for the inconsistent results.

There are several possible explanations for higher CHD risk when substituting total fat or SFA from dairy with the corresponding FA from meat. First, the intake of individual FA within the group of SFA differs between meat and dairy, and individual SFA seems to differ from one another with regards to biological function [28], oxidation rate [6] and association with lipid profile [18]. We observed a slightly higher contribution of C14:0, C15:0 and C17:0 to total SFA from dairy in comparison to SFA from meat, whereas the contribution of C18:0 and C22:0 was slightly lower. Within the even-chain FA, C12:0, C14:0 and C16:0 are considered to be detrimental to cardiovascular health through an effect on the LDL-receptor [9]. These even-chain FAs have also shown to have a detrimental effect on lipid profile [18]. On the other hand, dairy lipids including C15:0 and C17:0 may have anti-inflammatory properties [17], hypothetically leading to an improvement of the lipid profile.

A second hypothesis is that correlated components from meat and dairy sources might drive associations. For instance, meat contains heme iron which has been associated with higher CVD risk [8], whereas dairy components such as vitamin D, calcium, magnesium and potassium have been associated to a more beneficial cardiometabolic risk profile [27].

Finally, the dairy food matrix might have specific beneficial effects on cardiovascular health that are not completely explained by single components within the food. For instance, interactions between nutrients in the dairy matrix can be enhanced by dairy structures and processing methods. This may modify the metabolic effects of dairy consumption [33].

Strengths of this study include the large study population from a prospective cohort with a long follow-up time, and the direct comparison between FA from dairy and FA from meat through a substitution model. We have adjusted our results for a comprehensive set of confounders. Also, we performed sensitivity analyses to examine the robustness of our findings.

There are also limitations to address. First, we used an FFQ to measure our exposure which could have led to misclassification, although we expect this misclassification to be non-differential. Second, we used one dietary measurement at baseline to asses dietary intake. This influences the interpretation of our substitution model, as we did not investigate CHD risk for people that changed their diet over time. However, it is unlikely that a second dietary measurement would allow for examination of substitution of foods within the same person, as dietary habits have shown to be quite stable over time [21].

Another limitation is the potential for residual confounding in the substitution of dairy FA with meat FA, through a generally unhealthier lifestyle of participants with a high intake of meat, and a generally healthier lifestyle of participants with high intake of dairy.

Also, intake of total fat, SFA, MUFA and PUFA was highly correlated within the food group from which it was derived (dairy or meat), making it difficult to disentangle the effect of an individual FA from the effect of the other FAs in whole foods.

Finally, the results from this study should be interpreted with caution since we modelled substitution in an observational study. Actual substitution within individuals was thus not observed but statistically modelled. Also, causal inferences are inherently difficult to establish based on observational data.

In conclusion, our modelling suggests that substituting dairy SFA with meat SFA is associated with a higher risk of CHD, whereas substituting dairy MUFA or PUFA with meat is not. Since average SFA intake continues to exceed the 10en%/day that has been recommended for decades [7], identifying comparatively healthy fats depending on food source may help in improving nutritional advice. However, results of our substitution modelling need to be replicated in other cohorts with different background fat intakes, preferably with larger variation in the intake of MUFA and PUFA from dairy and meat. Also, results from substitution modelling should be confirmed in experimental studies before.

## Electronic supplementary material

Below is the link to the electronic supplementary material.

Supplementary material 1 (DOCX 74 KB)

## Abbreviations

BMI: Body mass index
CHD: Coronary heart disease
CPAI: Cambridge physical activity index
CVD: Cardiovascular disease
En%: Percentage of total energy intake
EPIC: European prospective investigation into cancer and nutrition
FA: Fatty acid
FFQ: Food frequency questionnaire
ICD: International classification of diseases
kcal: Kilocalorie
MORGEN: Monitoring project on risk factors for chronic diseases
MUFA: Mono-unsaturated fatty acids
PUFA: Poly-unsaturated fatty acids
SFA: Saturated fatty acids
SPSS: Statistical package for the social sciences
WHR: Waist-to-hip ratio
