# Supplementary material for: Fatty acids from dairy and meat and their association with risk of coronary heart disease
Source: Eur J Nutr. 2018 Aug 30;58(7):2639–47. doi: 10.1007/s00394-018-1811-1 (PMC6768909; doi:10.1007/s00394-018-1811-1)
Supplement: Supplementary file 1 — Supplementary material 1 (DOCX 74 KB) [file 394_2018_1811_MOESM1_ESM.docx]

**European Journal of Nutrition: Online supporting material**

**Fatty acids from dairy and meat and risk of coronary heart disease**

Linda E.T. Vissers, Jonna Rijksen, Jolanda M.A. Boer, W.M. Monique Verschuren, Yvonne T. van der Schouw, Ivonne Sluijs

- LV,JR,WV,YS,IS: Julius Center for Health Sciences and Primary Care, University Medical Center Utrecht, Utrecht, Utrecht University, the Netherlands.
- JB,WV: National institute for Public Health and the Environment (RIVM), Bilthoven, the Netherlands.

**Corresponding author:**

Ivonne Sluijs, PhD T: 0031 88 7568181 E: [I.Sluijs-2@umcutrecht.nl](mailto:I.Sluijs-2@umcutrecht.nl)

Julius Center for Health Sciences and Primary Care, University Medical Center Utrecht

STRT 6.131, PO Box 85500, 3508GA Utrecht, The Netherlands

**Supplemental Table 1. Whole food items that contribute to the meat and dairy product groups.**

| **Meat products** | Beef >5g fat, beef <5g fat, beefsteak, smoked beef, beef mince meat, half beef and half pork mince meat, smoked sausage, pork mince meat, shoulder ham, pork 4-14 g fat, pork >15g fat, bacon, salami, saveloy, liver sausage, pork liver, horse chicken meat, with skin, chicken meat without skin |
| --- | --- |
| **Dairy products** | Fresh farm milk, milk (full-fat, semi-skimmed, skimmed), buttermilk, chocolate milk, custard, porridge, curd cheese, ice cream, coffee milk (full fat, semi-skimmed, skimmed), coffee creamer, yogurt (full-fat, skimmed), yogurt drink, whipped cream, Gouda cheese, Edammer cheese 40+, Leyden cheese, brie 50+, trenta cheese, cheese on pizza |

**Supplemental Table 2. Baseline characteristics of 35,767 EPIC-NL cohort participants per tertile of dairy-derived saturated fat intake(in % of total energy intake minus energy from alcohol).**

|  | **En% of dairy-derived saturated fat** | | | | | | | |  |
| --- | --- | --- | --- | --- | --- | --- | --- | --- | --- |
|  | T1 (< 3,9) | |  | T2 (3,9-5,7) | |  | T3 (5,7-23,9) | |  |
| Participants (n) | 11922 | |  | 11923 | |  | 11922 | |  |
| Women (%) | 64,3 |  |  | 76,4 |  |  | 83,7 |  |  |
| Age (years) | 46 | ± | 12 | 50 | ± | 12 | 52 | ± | 11 |
| BMI (kg/m2) | 25,6 | ± | 4,0 | 25,7 | ± | 3,9 | 25,6 | ± | 4,0 |
| WHR | 0,83 | ± | 0,09 | 0,82 | ± | 0,09 | 0,81 | ± | 0,08 |
| High education level^a^ (%) | 17,9 |  |  | 20,1 |  |  | 23,0 |  |  |
| Light alcohol consumption^b^ (%) | 49,1 |  |  | 49,7 |  |  | 50,5 |  |  |
| Physically active (%) | 40,9 |  |  | 41,9 |  |  | 43,1 |  |  |
| Smoking |  |  |  |  |  |  |  |  |  |
| Former smoker (%) | 28,8 |  |  | 32,2 |  |  | 32,5 |  |  |
| Current smoker (%) | 34,9 |  |  | 28,2 |  |  | 28,0 |  |  |
| Hypertension (%) | 34,9 |  |  | 37,6 |  |  | 37,9 |  |  |
| Hypercholesterolemia (%) | 9,8 |  |  | 7,9 |  |  | 5,8 |  |  |
| Type 2 diabetes (%) | 1,1 |  |  | 1,2 |  |  | 1,7 |  |  |
|  |  |  |  |  |  |  |  |  |  |
| Energy intake (kcal/day) | 2114 | ± | 655 | 2059 | ± | 586 | 1989 | ± | 567 |
| Dairy products (g/day) | 283 | ± | 209 | 451 | ± | 243 | 570 | ± | 310 |
| Dairy fat (en%/day) |  |  |  |  |  |  |  |  |  |
| Total fat | 4,2 | ± | 1,4 | 7,4 | ± | 0,8 | 11,6 | ± | 2,6 |
| SFA | 2,7 | ± | 0,9 | 4,8 | ± | 0,5 | 7,4 | ± | 1,6 |
| MUFA | 1,2 | ± | 0,4 | 2,2 | ± | 0,3 | 3,5 | ± | 0,9 |
| PUFA | 0,2 | ± | 0,1 | 0,3 | ± | 0,1 | 0,5 | ± | 0,2 |
| Meat products (g/day) | 126 | ± | 61 | 107 | ± | 51 | 90 | ± | 50 |
| Meat fat (en%/day) |  |  |  |  |  |  |  |  |  |
| Total fat | 7,5 | ± | 3,8 | 6,5 | ± | 3,3 | 5,5 | ± | 3,2 |
| SFA | 2,9 | ± | 1,5 | 2,6 | ± | 1,3 | 2,2 | ± | 1,3 |
| MUFA | 3,5 | ± | 1,8 | 3,0 | ± | 1,5 | 2,6 | ± | 1,5 |
| PUFA | 0,9 | ± | 0,5 | 0,7 | ± | 0,4 | 0,6 | ± | 0,4 |
| Protein (en%/day) | 15,0 | ± | 2,4 | 16,1 | ± | 2,2 | 17,1 | ± | 2,2 |
| Carbohydrates (en%/day) | 48,4 | ± | 6,0 | 47,1 | ± | 5,3 | 45,1 | ± | 5,4 |
| Fat (en%/day) | 36,6 | ± | 5,6 | 36,8 | ± | 5,2 | 37,8 | ± | 5,1 |
| Trans fat (en%/day) | 1,5 | ± | 0,6 | 1,4 | ± | 0,5 | 1,4 | ± | 0,4 |
| Soft drinks (ml/day) | 131 | ± | 165 | 95 | ± | 125 | 75 | ± | 107 |
| Fruit and vegetables (g/day) | 383 | ± | 194 | 408 | ± | 182 | 403 | ± | 175 |
| Fish (g/day) | 10 | ± | 11 | 10 | ± | 11 | 10 | ± | 10 |
| Nuts and seeds (g/day) | 8 | ± | 13 | 7 | ± | 11 | 6 | ± | 9 |
| Coffee and tea (ml/day) | 809 | ± | 379 | 857 | ± | 335 | 885 | ± | 348 |
| Cholesterol ^c^(mg/day) | 208 | ± | 61 | 215 | ± | 56 | 230 | ± | 56 |
| Fibre^c^ (g/day) | 23 | ± | 5 | 24 | ± | 5 | 23 | ± | 4 |

Abbreviations: WHR, waist to hip ratio. En%, percentage of total energy intake; MUFA, mono unsaturated fatty acid; PUFA, poly unsaturated fatty acid; SFA, saturated fatty acid; Data is presented as mean ±SD or as percentage. ^a^ higher vocational education and university ^b^ <4.9 grams of alcohol per day ^c^ Energy adjusted.

**Supplemental Table 3. Baseline characteristics of 35,767 EPIC-NL cohort participants per tertile of meat-derived saturated fat intake (in % of total energy intake minus energy from alcohol).**

|  | **En% of meat-derived saturated fat** | | | | | | | | |
| --- | --- | --- | --- | --- | --- | --- | --- | --- | --- |
|  | T1 (<1.9) | |  | T2 (1.9 - 3.0) | |  | T3 (3.0 - 21.0) | | |
| Participants (n) | 11922 | |  | 11923 | |  | 11922 | |  |
| Women (%) | 82,2 |  |  | 72,8 |  |  | 69,5 |  |  |
| Age (years) | 50 | ± | 12 | 49 | ± | 12 | 49 | ± | 11 |
| BMI (kg/m2) | 24,8 | ± | 3,7 | 25,7 | ± | 3,9 | 26,5 | ± | 4,1 |
| WHR | 0,80 | ± | 0,08 | 0,82 | ± | 0,09 | 0,84 | ± | 0,09 |
| High education level^a^ (%) | 27,2 |  |  | 19,8 |  |  | 14,0 |  |  |
| Light alcohol consumption^b^ (%) | 54,8 |  |  | 48,5 |  |  | 46,0 |  |  |
| Physically active (%) | 43,1 |  |  | 43,1 |  |  | 39,8 |  |  |
| Smoking |  |  |  |  |  |  |  |  |  |
| Former smoker (%) | 61,8 |  |  | 61,1 |  |  | 30,7 |  |  |
| Current smoker (%) | 26,1 |  |  | 29,6 |  |  | 35,4 |  |  |
| Hypertension (%) | 34,5 |  |  | 36,4 |  |  | 39,5 |  |  |
| Hypercholesterolemia (%) | 7,2 |  |  | 7,5 |  |  | 8,8 |  |  |
| Type 2 diabetes (%) | 1,1 |  |  | 1,2 |  |  | 1,7 |  |  |
|  |  |  |  |  |  |  |  |  |  |
| Energy intake (kcal/day) | 1991 | ± | 601 | 2116 | ± | 613 | 2054 | ± | 597 |
| Dairy products (g/day) | 495 | ± | 309 | 447 | ± | 274 | 361 | ± | 247 |
| Dairy fat (en%/day) |  |  |  |  |  |  |  |  |  |
| Total fat | 8,8 | ± | 3,8 | 7,6 | ± | 3,2 | 6,8 | ± | 3,2 |
| SFA | 5,6 | ± | 2,4 | 4,9 | ± | 2,0 | 4,4 | ± | 2,0 |
| MUFA | 2,7 | ± | 1,2 | 2,3 | ± | 1,0 | 2,0 | ± | 1,0 |
| PUFA | 0,4 | ± | 0,2 | 0,3 | ± | 0,2 | 0,3 | ± | 0,2 |
| Meat products (g/day) | 58 | ± | 33 | 112 | ± | 36 | 153 | ± | 50 |
| Meat fat (en%/day) |  |  |  |  |  |  |  |  |  |
| Total fat | 2,9 | ± | 1,2 | 6,1 | ± | 0,9 | 10,4 | ± | 2,6 |
| SFA | 1,2 | ± | 0,5 | 2,4 | ± | 0,3 | 4,1 | ± | 1,0 |
| MUFA | 1,4 | ± | 0,6 | 2,9 | ± | 0,4 | 4,9 | ± | 1,2 |
| PUFA | 0,3 | ± | 0,2 | 0,7 | ± | 0,2 | 1,2 | ± | 0,4 |
| Protein (en%/day) | 15,4 | ± | 2,4 | 16,1 | ± | 2,3 | 16,8 | ± | 2,4 |
| Carbohydrates (en%/day) | 49,8 | ± | 5,4 | 47,1 | ± | 4,8 | 43,7 | ± | 5,1 |
| Fat (en%/day) | 34,8 | ± | 5,3 | 36,8 | ± | 4,7 | 39,5 | ± | 4,9 |
| Trans fat (en%/day) | 1,4 | ± | 0,5 | 1,5 | ± | 0,5 | 1,5 | ± | 0,5 |
| Soft drinks (ml/day) | 88 | ± | 128 | 105 | ± | 136 | 108 | ± | 144 |
| Fruit and vegetables (g/day) | 440 | ± | 198 | 398 | ± | 176 | 357 | ± | 168 |
| Fish (g/day) | 11 | ± | 13 | 10 | ± | 10 | 9 | ± | 9 |
| Nuts and seeds (g/day) | 8 | ± | 13 | 7 | ± | 11 | 5 | ± | 9 |
| Coffee and tea (ml/day) | 847 | ± | 367 | 849 | ± | 341 | 855 | ± | 360 |
| Cholesterol^c^ (mg/day) | 195 | ± | 57 | 218 | ± | 53 | 241 | ± | 56 |
| Fibre^c^ (g/day) | 24 | ± | 5 | 23 | ± | 5 | 22 | ± | 5 |

Abbreviations: WHR, waist to hip ratio. En%, percentage of total energy intake; MUFA, mono unsaturated fatty acid; PUFA, poly unsaturated fatty acid; SFA, saturated fatty acid; Data is presented as mean ±SD or as percentage. ^a^ higher vocational education and university ^b^ <4.9 grams of alcohol per day ^c^ Energy adjusted.

**Supplemental Table 4. Baseline characteristics of 35,767 EPIC-NL cohort participants per tertile of dairy-derived mono-unsaturated fat intake(in % of total energy intake minus energy from alcohol).**

|  | **En% of dairy-derived mono-unsaturated fat** | | | | | | | | |
| --- | --- | --- | --- | --- | --- | --- | --- | --- | --- |
|  | T1 (< 1.8) | |  | T2 (1.8 - 2.7) | |  | T3 (2.7 - 11.9) | | |
| Participants (n) | 11922 | |  | 11923 | |  | 11922 | |  |
| Women (%) | 64,1 |  |  | 77,2 |  |  | 83,2 |  |  |
| Age (years) | 46 | ± | 12 | 50 | ± | 12 | 52 | ± | 11 |
| BMI (kg/m2) | 25,6 | ± | 4,0 | 25,7 | ± | 3,9 | 25,6 | ± | 4,0 |
| WHR | 0,83 | ± | 0,09 | 0,82 | ± | 0,09 | 0,81 | ± | 0,08 |
| High education level^a^ (%) | 17,8 |  |  | 20,2 |  |  | 23,0 |  |  |
| Light alcohol consumption^b^ (%) | 49,9 |  |  | 49,9 |  |  | 49,5 |  |  |
| Physically active (%) | 40,9 |  |  | 41,9 |  |  | 43,1 |  |  |
| Smoking |  |  |  |  |  |  |  |  |  |
| Former smoker (%) | 28,6 |  |  | 32,4 |  |  | 32,7 |  |  |
| Current smoker (%) | 34,5 |  |  | 28,3 |  |  | 28,3 |  |  |
| Hypertension (%) | 34,2 |  |  | 38,4 |  |  | 37,7 |  |  |
| Hypercholesterolemia (%) | 9,6 |  |  | 8,0 |  |  | 5,9 |  |  |
| Type 2 diabetes (%) | 1,1 |  |  | 1,3 |  |  | 1,6 |  |  |
|  |  |  |  |  |  |  |  |  |  |
| Energy intake (kcal/day) | 2118 | ± | 652 | 2050 | ± | 584 | 1994 | ± | 573 |
| Dairy products (g/day) | 294 | ± | 215 | 454 | ± | 249 | 556 | ± | 311 |
| Dairy fat (en%/day) |  |  |  |  |  |  |  |  |  |
| Total fat | 4,2 | ± | 1,4 | 7,4 | ± | 0,9 | 11,5 | ± | 2,6 |
| SFA | 2,8 | ± | 0,9 | 4,8 | ± | 0,6 | 7,4 | ± | 1,7 |
| MUFA | 1,2 | ± | 0,4 | 2,2 | ± | 0,3 | 3,6 | ± | 0,8 |
| PUFA | 0,2 | ± | 0,1 | 0,3 | ± | 0,1 | 0,5 | ± | 0,2 |
| Meat products (g/day) | 126 | ± | 60 | 107 | ± | 51 | 90 | ± | 51 |
| Meat fat (en%/day) |  |  |  |  |  |  |  |  |  |
| Total fat | 7,5 | ± | 3,7 | 6,5 | ± | 3,3 | 5,5 | ± | 3,2 |
| SFA | 2,9 | ± | 1,5 | 2,6 | ± | 1,3 | 2,2 | ± | 1,3 |
| MUFA | 3,5 | ± | 1,8 | 3,0 | ± | 1,5 | 2,6 | ± | 1,5 |
| PUFA | 0,9 | ± | 0,5 | 0,7 | ± | 0,4 | 0,6 | ± | 0,4 |
| Protein (en%/day) | 15,0 | ± | 2,3 | 16,1 | ± | 2,2 | 17,2 | ± | 2,3 |
| Carbohydrates (en%/day) | 48,5 | ± | 5,9 | 47,1 | ± | 5,3 | 45,0 | ± | 5,3 |
| Fat (en%/day) | 36,5 | ± | 5,5 | 36,8 | ± | 5,2 | 37,9 | ± | 5,1 |
| Trans fat (en%/day) | 1,5 | ± | 0,6 | 1,4 | ± | 0,5 | 1,4 | ± | 0,4 |
| Soft drinks (ml/day) | 132 | ± | 164 | 94 | ± | 125 | 75 | ± | 108 |
| Fruit and vegetables (g/day) | 382 | ± | 194 | 409 | ± | 181 | 404 | ± | 176 |
| Fish (g/day) | 10 | ± | 11 | 10 | ± | 11 | 10 | ± | 10 |
| Nuts and seeds (g/day) | 8 | ± | 13 | 7 | ± | 11 | 6 | ± | 9 |
| Coffee and tea (ml/day) | 807 | ± | 378 | 861 | ± | 333 | 882 | ± | 351 |
| Cholesterol^c^ (mg/day) | 208 | ± | 61 | 216 | ± | 56 | 230 | ± | 56 |
| Fibre^c^ (g/day) | 23 | ± | 5 | 24 | ± | 5 | 23 | ± | 5 |

Abbreviations: WHR, waist to hip ratio. En%, percentage of total energy intake; MUFA, mono unsaturated fatty acid; PUFA, poly unsaturated fatty acid; SFA, saturated fatty acid; Data is presented as mean ±SD or as percentage. ^a^ higher vocational education and university ^b^ <4.9 grams of alcohol per day ^c^ Energy adjusted.

**Supplemental Table 5. Baseline characteristics of 35,767 EPIC-NL cohort participants per tertile of meat-derived mono-unsaturated fat intake (% of total energy intake minus energy from alcohol).**

|  | **En% of meat-derived mono-unsaturated fat** | | | | | | | | |
| --- | --- | --- | --- | --- | --- | --- | --- | --- | --- |
|  | T1 (< 2.2) | |  | T2 (2.2 - 3.6) | |  | T3 (3.6 - 25.5) | | |
| Participants (n) | 11922 | |  | 11923 | |  | 11922 | |  |
| Women (%) | 82,1 |  |  | 73,3 |  |  | 69,0 |  |  |
| Age (years) | 50 | ± | 12 | 49 | ± | 12 | 49 | ± | 11 |
| BMI (kg/m2) | 24,8 | ± | 3,7 | 25,7 | ± | 3,9 | 26,5 | ± | 4,1 |
| WHR | 0,80 | ± | 0,08 | 0,82 | ± | 0,09 | 0,84 | ± | 0,09 |
| High education level^a^ (%) | 27,3 |  |  | 19,7 |  |  | 14,0 |  |  |
| Light alcohol consumption^b^ (%) | 54,6 |  |  | 48,9 |  |  | 45,8 |  |  |
| Physically active (%) | 43,0 |  |  | 42,8 |  |  | 40,0 |  |  |
| Smoking |  |  |  |  |  |  |  |  |  |
| Former smoker (%) | 31,8 |  |  | 31,0 |  |  | 30,8 |  |  |
| Current smoker (%) | 26,2 |  |  | 29,5 |  |  | 35,5 |  |  |
| Hypertension (%) | 34,4 |  |  | 36,7 |  |  | 39,3 |  |  |
| Hypercholesterolemia (%) | 7,2 |  |  | 7,5 |  |  | 8,8 |  |  |
| Type 2 diabetes (%) | 1,1 |  |  | 1,2 |  |  | 1,7 |  |  |
|  |  |  |  |  |  |  |  |  |  |
| Energy intake (kcal/day) | 1991 | ± | 600 | 2105 | ± | 609 | 2066 | ± | 603 |
| Dairy products (g/day) | 496 | ± | 308 | 446 | ± | 273 | 362 | ± | 248 |
| Dairy fat (en%/day) |  |  |  |  |  |  | 7 |  | 3 |
| Total fat | 8,8 | ± | 3,8 | 7,6 | ± | 3,2 | 7 |  | 3 |
| SFA | 5,7 | ± | 2,4 | 4,9 | ± | 2,0 | 4,4 | ± | 2,0 |
| MUFA | 2,7 | ± | 1,2 | 2,3 | ± | 1,0 | 2,0 | ± | 1,0 |
| PUFA | 0,4 | ± | 0,2 | 0,3 | ± | 0,2 | 0,3 | ± | 0,2 |
| Meat products (g/day) | 59 | ± | 33 | 111 | ± | 36 | 154 | ± | 50 |
| Meat fat (en%/day) |  |  |  |  |  |  |  |  |  |
| Total fat | 2,9 | ± | 1,2 | 6,1 | ± | 0,9 | 10 | ± | 3 |
| SFA | 1,2 | ± | 0,5 | 2,4 | ± | 0,3 | 4 | ± | 1 |
| MUFA | 1,4 | ± | 0,6 | 2,9 | ± | 0,4 | 4,9 | ± | 1,2 |
| PUFA | 0,3 | ± | 0,2 | 0,7 | ± | 0,2 | 1,2 | ± | 0,4 |
| Protein (en%/day) | 15,4 | ± | 2,4 | 16,1 | ± | 2,3 | 16,7 | ± | 2,4 |
| Carbohydrates (en%/day) | 49,7 | ± | 5,4 | 47,1 | ± | 4,8 | 43,7 | ± | 5,1 |
| Fat (en%/day) | 34,8 | ± | 5,3 | 36,8 | ± | 4,7 | 39,5 | ± | 4,8 |
| Trans fat (en%/day) | 1,4 | ± | 0,5 | 1,5 | ± | 0,5 | 1,5 | ± | 0,5 |
| Soft drinks (ml/day) | 87 | ± | 127 | 105 | ± | 135 | 109 | ± | 146 |
| Fruit and vegetables (g/day) | 440 | ± | 198 | 397 | ± | 176 | 357 | ± | 168 |
| Fish (g/day) | 11 | ± | 13 | 10 | ± | 10 | 9 | ± | 9 |
| Nuts and seeds (g/day) | 8 | ± | 13 | 7 | ± | 11 | 5 | ± | 9 |
| Coffee and tea (ml/day) | 848 | ± | 362 | 848 | ± | 344 | 855 | ± | 361 |
| Cholesterol^c^ (mg/day) | 195 | ± | 57 | 218 | ± | 53 | 241 | ± | 56 |
| Fibre^c^ (g/day) | 24 | ± | 5 | 23 | ± | 5 | 22 | ± | 5 |

Abbreviations: WHR, waist to hip ratio. En%, percentage of total energy intake; MUFA, mono unsaturated fatty acid; PUFA, poly unsaturated fatty acid; SFA, saturated fatty acid; Data is presented as mean ±SD or as percentage. ^a^ higher vocational education and university ^b^ <4.9 grams of alcohol per day ^c^ Energy adjusted.

**Supplemental Table 6. Baseline characteristics of 35,767 EPIC-NL cohort participants per tertile of dairy-derived poly-unsaturated fat intake (in % of total energy intake minus energy from alcohol).**

|  | **En% of dairy-derived poly-unsaturated fat** | | | | | | | | |
| --- | --- | --- | --- | --- | --- | --- | --- | --- | --- |
|  | T1 (< 0.2) | |  | T2 (0.2-0.4) | |  | T3 (0.4-3.0) | |  |
| Participants (n) | 11922 | |  | 11923 | |  | 11922 | |  |
| Women (%) | 64,9 |  |  | 76,8 |  |  | 82,7 |  |  |
| Age (years) | 46 | ± | 12 | 50 | ± | 11 | 52 | ± | 11 |
| BMI (kg/m2) | 25,6 | ± | 4,0 | 25,7 | ± | 4,0 | 25,7 | ± | 4,0 |
| WHR | 0,83 | ± | 0,09 | 0,82 | ± | 0,09 | 0,82 | ± | 0,08 |
| High education level^a^ (%) | 18,5 |  |  | 20,1 |  |  | 21,7 |  |  |
| Light alcohol consumption^b^ (%) | 48,4 |  |  | 49,4 |  |  | 51,5 |  |  |
| Physically active (%) | 41,8 |  |  | 42,3 |  |  | 41,8 |  |  |
| Smoking |  |  |  |  |  |  |  |  |  |
| Former smoker (%) | 28,8 |  |  | 32,5 |  |  | 32,3 |  |  |
| Current smoker (%) | 33,6 |  |  | 28,6 |  |  | 28,9 |  |  |
| Hypertension (%) | 33,6 |  |  | 37,5 |  |  | 39,2 |  |  |
| Hypercholesterolemia (%) | 8,3 |  |  | 7,6 |  |  | 7,6 |  |  |
| Type 2 diabetes (%) | 0,9 |  |  | 1,3 |  |  | 1,7 |  |  |
|  |  |  |  |  |  |  |  |  |  |
| Energy intake (kcal/day) | 2134 | ± | 654 | 2062 | ± | 584 | 1965 | ± | 564 |
| Dairy products (g/day) | 331 | ± | 245 | 464 | ± | 271 | 509 | ± | 299 |
| Dairy fat (en%/day) |  |  |  |  |  |  |  |  |  |
| Total fat | 4,4 | ± | 1,6 | 7,6 | ± | 1,4 | 11,1 | ± | 3,0 |
| SFA | 2,9 | ± | 1,1 | 5,0 | ± | 1,0 | 7,1 | ± | 2,0 |
| MUFA | 1,3 | ± | 0,5 | 2,3 | ± | 0,4 | 3,4 | ± | 1,0 |
| PUFA | 0,2 | ± | 0,1 | 0,3 | ± | 0,0 | 0,5 | ± | 0,2 |
| Meat products (g/day) | 126 | ± | 60 | 107 | ± | 51 | 91 | ± | 51 |
| Meat fat (en%/day) |  |  |  |  |  |  |  |  |  |
| Total fat | 7,4 | ± | 3,7 | 6,5 | ± | 3,3 | 5,5 | ± | 3,3 |
| SFA | 2,9 | ± | 1,4 | 2,6 | ± | 1,3 | 2,2 | ± | 1,3 |
| MUFA | 3,5 | ± | 1,8 | 3,0 | ± | 1,6 | 2,6 | ± | 1,5 |
| PUFA | 0,9 | ± | 0,5 | 0,7 | ± | 0,4 | 0,6 | ± | 0,4 |
| Protein (en%/day) | 15,1 | ± | 2,3 | 16,1 | ± | 2,2 | 17,1 | ± | 2,3 |
| Carbohydrates (en%/day) | 48,4 | ± | 5,8 | 47,0 | ± | 5,3 | 45,2 | ± | 5,5 |
| Fat (en%/day) | 36,5 | ± | 5,5 | 36,9 | ± | 5,2 | 37,7 | ± | 5,2 |
| Trans fat (en%/day) | 1,5 | ± | 0,6 | 1,4 | ± | 0,5 | 1,4 | ± | 0,4 |
| Soft drinks (ml/day) | 132 | ± | 164 | 93 | ± | 125 | 75 | ± | 108 |
| Fruit and vegetables (g/day) | 385 | ± | 193 | 406 | ± | 182 | 403 | ± | 177 |
| Fish (g/day) | 10 | ± | 11 | 10 | ± | 11 | 10 | ± | 10 |
| Nuts and seeds (g/day) | 8 | ± | 13 | 7 | ± | 11 | 6 | ± | 9 |
| Coffee and tea (ml/day) | 800 | ± | 378 | 858 | ± | 335 | 893 | ± | 347 |
| Cholesterol^c^ (mg/day) | 209 | ± | 61 | 218 | ± | 56 | 227 | ± | 57 |
| Fibre^c^ (g/day) | 23 | ± | 5 | 23 | ± | 5 | 23 | ± | 5 |

Abbreviations: WHR, waist to hip ratio. En%, percentage of total energy intake; MUFA, mono unsaturated fatty acid; PUFA, poly unsaturated fatty acid; SFA, saturated fatty acid; Data is presented as mean ±SD or as percentage. ^a^ higher vocational education and university ^b^ <4.9 grams of alcohol per day ^c^ Energy adjusted.

**Supplemental Table 7. Baseline characteristics of 35,767 EPIC-NL cohort participants per tertile of meat-derived poly-unsaturated fat intake (in % of total energy intake minus energy from alcohol).**

|  | **En% of meat-derived poly-unsaturated fat** | | | | | | | |  |
| --- | --- | --- | --- | --- | --- | --- | --- | --- | --- |
|  | T1 (<0.5) | |  | T2 (0.5-0.9) | |  | T3 (0.9-4.7) | |  |
| Participants (n) | 11922 | |  | 11923 | |  | 11922 | |  |
| Women (%) | 82,1 |  |  | 73,5 |  |  | 69,0 |  |  |
| Age (years) | 50 | ± | 12 | 49 | ± | 12 | 49 | ± | 11 |
| BMI (kg/m2) | 24,8 | ± | 3,7 | 25,6 | ± | 3,8 | 26,5 | ± | 4,2 |
| WHR | 0,80 | ± | 0,08 | 0,82 | ± | 0,09 | 0,84 | ± | 0,09 |
| High education level^a^ (%) | 27,5 |  |  | 20,4 |  |  | 13,0 |  |  |
| Light alcohol consumption^b^ (%) | 54,1 |  |  | 48,0 |  |  | 47,2 |  |  |
| Physically active (%) | 42,7 |  |  | 43,0 |  |  | 40,2 |  |  |
| Smoking |  |  |  |  |  |  |  |  |  |
| Former smoker (%) | 31,7 |  |  | 31,5 |  |  | 30,4 |  |  |
| Current smoker (%) | 25,7 |  |  | 29,7 |  |  | 35,7 |  |  |
| Hypertension (%) | 34,7 |  |  | 36,4 |  |  | 39,3 |  |  |
| Hypercholesterolemia (%) | 7,2 |  |  | 7,7 |  |  | 8,6 |  |  |
| Type 2 diabetes (%) | 1,1 |  |  | 1,2 |  |  | 1,7 |  |  |
|  |  |  |  |  |  |  |  |  |  |
| Energy intake (kcal/day) | 1978 | ± | 591 | 2102 | ± | 617 | 2081 | ± | 602 |
| Dairy products (g/day) | 493 | ± | 306 | 445 | ± | 277 | 365 | ± | 247 |
| Dairy fat (en%/day) |  |  |  |  |  |  |  |  |  |
| Total fat | 8,8 | ± | 3,7 | 7,7 | ± | 3,2 | 6,8 | ± | 3,1 |
| SFA | 5,6 | ± | 2,4 | 4,9 | ± | 2,1 | 4,4 | ± | 2,0 |
| MUFA | 2,7 | ± | 1,2 | 2,3 | ± | 1,0 | 2,0 | ± | 1,0 |
| PUFA | 0,4 | ± | 0,2 | 0,3 | ± | 0,2 | 0,3 | ± | 0,2 |
| Meat products (g/day) | 61 | ± | 35 | 111 | ± | 39 | 152 | ± | 50 |
| Meat fat (en%/day) |  |  |  |  |  |  |  |  |  |
| Total fat | 3,0 | ± | 1,4 | 6,2 | ± | 1,2 | 10,2 | ± | 2,7 |
| SFA | 1,2 | ± | 0,6 | 2,5 | ± | 0,5 | 4,0 | ± | 1,1 |
| MUFA | 1,4 | ± | 0,7 | 2,9 | ± | 0,6 | 4,8 | ± | 1,3 |
| PUFA | 0,3 | ± | 0,1 | 0,7 | ± | 0,1 | 1,3 | ± | 0,4 |
| Protein (en%/day) | 15,6 | ± | 2,4 | 16,1 | ± | 2,4 | 16,6 | ± | 2,4 |
| Carbohydrates (en%/day) | 49,6 | ± | 5,5 | 47,1 | ± | 5,0 | 44,0 | ± | 5,2 |
| Fat (en%/day) | 34,8 | ± | 5,3 | 36,8 | ± | 4,8 | 39,5 | ± | 4,9 |
| Trans fat (en%/day) | 1,3 | ± | 0,5 | 1,5 | ± | 0,5 | 1,5 | ± | 0,5 |
| Soft drinks (ml/day) | 87 | ± | 128 | 105 | ± | 134 | 110 | ± | 145 |
| Fruit and vegetables (g/day) | 442 | ± | 196 | 399 | ± | 178 | 354 | ± | 167 |
| Fish (g/day) | 11 | ± | 12 | 10 | ± | 10 | 9 | ± | 9 |
| Nuts and seeds (g/day) | 8 | ± | 12 | 7 | ± | 11 | 6 | ± | 9 |
| Coffee and tea (ml/day) | 847 | ± | 358 | 850 | ± | 350 | 854 | ± | 360 |
| Cholesterol^c^ (mg/day) | 196 | ± | 57 | 219 | ± | 54 | 239 | ± | 57 |
| Fibre^c^ (g/day) | 24 | ± | 5 | 23 | ± | 5 | 22 | ± | 5 |

Abbreviations: WHR, waist to hip ratio. En%, percentage of total energy intake; MUFA, mono unsaturated fatty acid; PUFA, poly unsaturated fatty acid; SFA, saturated fatty acid; Data is presented as mean ±SD or as percentage. ^a^ higher vocational education and university ^b^ <4.9 grams of alcohol per day ^c^ Energy adjusted.

**Supplemental Figure 1. Dietary sources of SFA, MUFA and PUFA among 35,767 EPIC-NL cohort participants.**

Median SFA, PUFA and MUFA intake from different sources, expressed as a percentage of total SFA, PUFA and MUFA intake.
